# Neuropharmacological Effects of Mesaconitine: Evidence from Molecular and Cellular Basis of Neural Circuit

**DOI:** 10.1155/2020/8814531

**Published:** 2020-08-21

**Authors:** Zhihui Sun, Limin Yang, Lihong Zhao, Ranji Cui, Wei Yang

**Affiliations:** ^1^Cultivation Base of State Key Laboratory for Ecological Restoration and Ecosystem Management of Jilin Province and Ministry of Science and Technology, College of Chinese Medicinal Materials, Jilin Agricultural University, 130118, Changchun, Jilin, China; ^2^Jilin Provincial Key Laboratory on Molecular and Chemical Genetic, The Second Hospital of Jilin University, Changchun, China

## Abstract

Mesaconitine (MA), a diester-diterpenoid alkaloid in aconite roots, is considered to be one of the most important bioactive ingredients. In this review, we summarized its neuropharmacological effects, including analgesic effects and antiepileptiform effects. Mesaconitine can act on the central noradrenergic system and the serotonin system; behaving like the norepinephrine reuptake inhibitors and tricyclic antidepressants that increase norepinephrine levels in stress-induced depression. Therefore, the possible perspectives for future studies on the depression of MA were also discussed as well. The pharmacological effect of MA on depression is worthy of further study.

## 1. Introduction

Many antidepressants have been developed based on the catecholamine deficiency hypothesis, but these drugs cannot meet people's needs. The latency period in antidepressant efficacy is a problem in Major Depressive Disorder (MDD) treatment because the depressive states are often connected with a higher risk of suicide [1, 2]. Representative antidepressants like NA reuptake inhibitors (NRIs) and selective serotonin reuptake inhibitors (SSRIs) require long-term therapy [3, 4]. Besides, only about 50% of MDD patients who received currently available antidepressants (AD) showed complete remission, including several drug trials with or without concurrent psychotherapy, but up to 80% of patients showed partial response [5]. Tricyclic antidepressants may have cardiotoxicity and atropine-like side effects [6]. Many new antidepressants are variants of classic antidepressants and have similar limitations [7, 8]. Therefore, there is a need to develop better antidepressants.

**Figure 1:**
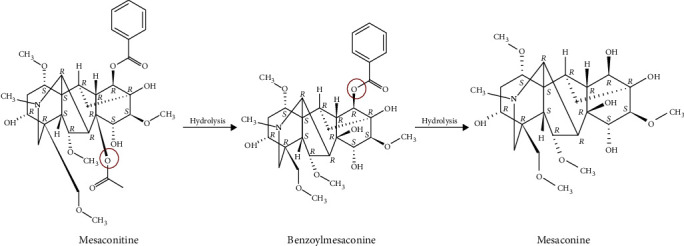
Absolute stereochemistry of mesaconitine and catabolite.

Mesaconitine (MA) is a predominant and representative component of alkaloids contained in the plant of the genera Aconitum [9]. MA possesses multiple pharmacological activities, such as vaso-relaxing effects [10–12], analgesic effects [13], and antiepileptiform effects [14]. Currently, only Nesterova et al. reported that MA possesses antidepressant activity [15]. However, the mechanisms of MA in analgesia and antiepileptiform effects are similar to that of antidepressants. The biological and pharmaceutical properties of MA can be improved by structural modification (Figure 1). So, the pharmacological effect of MA on depression is worthy of further study. In this review, we summarized the analgesic effects and antiepileptic effects. Besides, the possible perspectives for future studies on the depression of MA were also discussed as well.

## 2. Neuropharmacological Effects of MA

### 2.1. Analgesic Effects of MA

**Figure 2:**
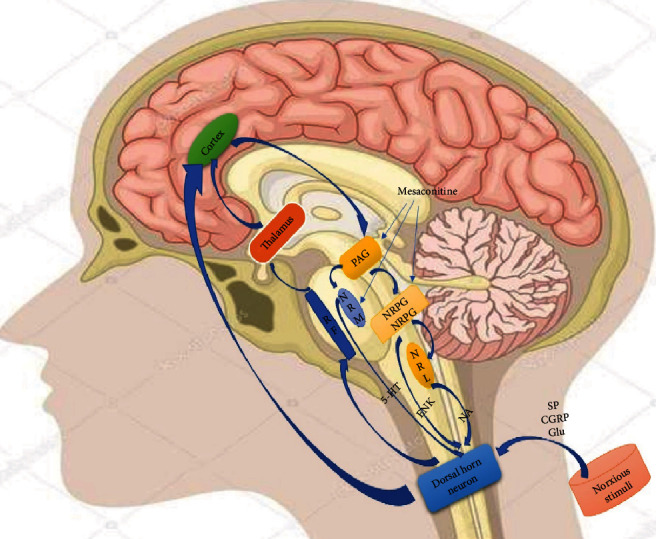
Schematic drawing of the nociceptive system with ascending and descending fibers. RF: reticular formation; PAG: periaqueductal gray; NRM: nucleus raphe magnus; NRGC: nucleus reticularis gigantocellularis; NRPG: nucleus reticularis paragigantocellularis; NRL: nucleus reticularis lateralis; 5-HT: 5-hydroxytryptamine; ENK: enkephalin; NA: noradrenaline.

Mesaconitine (MA) possesses antinociceptive activity in nociceptive test models, such as writhing and tail immersion test [16–18]. Also, several components of Fuzi (genera Aconitum) possess analgesic effects, such as hypaconitine, fuziline, neoline, aconitine, songorine, and mesaconitine [18–20]. Benzoylmesaconine, hydrolyte of mesaconitine [21], possesses antinociceptive action in hyperalgesic rats as well [22]. Among these alkaloids, mesaconitine exhibited the strongest analgesic effects [23]. Mesaconitine exerted analgesic effects via the periaqueductal gray (PAG), the nucleus reticularis gigantocellularis (NRGC), the lumbar enlargement, the nucleus raphe magnus (NRM), and the nucleus reticularis paragigantocellularis (NRPG) [17, 22, 24]. Microinjection of mesaconitine into the NRPG, NRM, and PAG produced dose-dependent analgesic activity; The analgesic effect of MA in NRM was more potent and sensitive than PAG and NRPG. [22]. Analgesic activity of the benzoylmesaconine (BM) may be through the activation of the NRM [22]. In the medulla oblongata, NRM is involved in the serotonergic descending inhibitory systems and NRPG acts in noradrenergic descending inhibitory systems. In the mesencephalon, PAG is involved in the descending pain inhibitory systems. Analgesic action of MA and BM appears to be through a descending serotonin system. MA promoted the turnover rate of norepinephrine in the brain stem and spinal cord. Norepinephrine activated adenylate cyclase through *β*-adrenoceptors, thereby significantly increased the level of cyclic adenosine monophosphate (cAMP), which enhanced the analgesic activity of MA [25, 26]. Murayama et al. reported that in isolated guinea-pig vas deferens, MA promoted the release of norepinephrine through excitatory sympathetic nerve fibers. Its analgesic effect may be the result of the release of noradrenaline from nerve endings and increased receptor sensitivity [26]. The analgesic effect of MA was enhanced by the injection of norepinephrine or isoproterenol intracerebroventricularly (i.c.v.) and attenuated by *β*-adrenoceptors antagonist [17, 26]. Therefore, the analgesic effect of MA seems to be through the activation of the noradrenergic system and serotonergic descending systems. This information is outlined in Table 1 and Figure 2.

Some studies have shown that the aromatic ester group of MA bound to site 2 of Na^+^channels, resulting in sodium ion influx, causing neuronal depolarization and ultimately inhibiting the transmission of pain [18]. Besides, MA has the highest concentration in aconitine-type alkaloids of water extract of Radix Aconiti Carmichaeli (Chuan Wu) as quantified by high-performance liquid chromatography; the analgesic and anti-inflammatory activity of aqueous extracts may be due to high concentration of MA [13]. Heishunpian, Baifupian, and Yan-Fuzi are processed products of Fuzi. Interestingly, compared to Heishunpian and Baifupian, Yan-Fuzi possesses less toxic and antinociceptive activity of Yan-Fuzi is similar to crude Fuzi [27], which may be differences in processing methods that resulted in different alkaloid contents [28].

### 2.2. Antiepileptiform Effects of MA

From the above statements, we already know that mesaconitine exerts analgesic effects by stimulating the noradrenergic system. Mesaconitine also inhibited epileptic field potentials through *α*-adrenoceptors in a concentration-dependent manner. Important components of epileptiform discharge include presynaptic fiber peaks, the first postsynaptic population spike, and succeeding spikes, which define epileptiform activity. Stimulation-triggered epileptiform activity (a nominal Mg^2+^-free perfusate) and spontaneous epileptiform activity (a nominal Mg^2+^-free perfusate with elevated K+ concentration (5 mM)) are inhibited by MA (30 nM), which was antagonized by the *α*-adrenoceptors antagonist yohimbine (YOH) [14]. However, MA (300 nM and 1 *μ*M) completely inhibited trigger-induced epileptiform activity and yohimbine cannot antagonize the inhibitory effect of MA [14]. These results indicated that MA (30 nM) activated the *α*-adrenoceptors when it inhibited experimentally induced epilepsy-like activity in the hippocampus.

**Figure 3:**
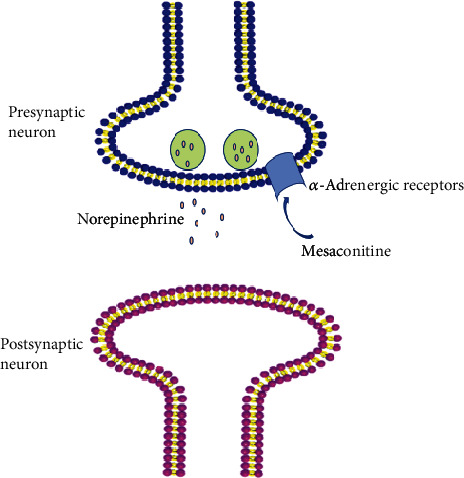
Schematic drawing of the antiepileptiform mechanism of mesaconitine on a noradrenergic neuron in the pyramidal stratum of the hippocampus.

Norepinephrine is believed to have both a convulsive and anticonvulsant effect depending on the receptors that are activated [29]. The hippocampus receives a diffuse projection of norepinephrine fibers from the locus coeruleus, and the activation of noradrenergic afferents affect hippocampal neuron activity [24]. The activation of alpha-adrenergic receptors reduced epileptiform discharges, whereas activation of beta-adrenergic receptors increased epileptiform discharges in the hippocampus. The rate of discharges induced by either picrotoxin or elevated extracellular potassium ([K+]_o_) was slowed by NA(≥10 *μ*M) [29]. Both *α*_1_-adrenergic receptors agonists (phenylephrine) and *α*_2_-adrenergic receptors antagonists (yohimbine) slowed epileptiform discharge rates [29]. However, some scholars have reported that the anticonvulsant activity of norepinephrine was mediated by *α*_2_-adrenergic receptors and the *α*_2_-selective agonist inhibited epileptiform discharges [30, 31]. The difference in the antiepileptiform mechanism of NA may be due to the different brain parts and concentrations of applied drugs. Consistently, the antiepileptiform effect is exerted by *α*-adrenergic receptors, and it is yet to be proven which specific *α*-adrenergic receptors have worked. It has been reported that mesaconitine induced contractions of the guinea-pig vas deferens by an enhanced neuronal release of noradrenaline [32]. So, MA (like the agonist) may act on *α*-adrenergic receptors to promote the release of norepinephrine and exert antiepileptiform effects (Figure 3). Besides, the specific molecular mechanism of antiepileptiform effects of MA needs further clarification.

### 2.3. Antidepressant Effects of MA

Our previous studies have demonstrated that Fuzi total alkaloids exerted anticonvulsant and antidepressant effects [33, 34]. Also, the antiepileptiform activity of MA has been reported. Some scholars have demonstrated the antidepressant effect of MA, possibly due to the altering of sensitivity to serotonin [15]. It is similar to the mechanism of the analgesic effects of MA. There are other possible reasons to support further the study of the pharmacological effect of MA on depression.

First, at least half of patients with chronic pain and itching are accompanied by depression and anxiety; chronic pain and itching can be found in as much as 60% of depressed patients. Tricyclic antidepressants have analgesic and antipruritic effects. Drugs treating psychosis can be used for analgesia, and analgesic drugs can also be used for depression [35]. We already knew that MA has an analgesic effect. Second, some scholars have demonstrated that the locus coeruleus noradrenergic neuron *α*_2_-adrenergic receptors are functionally blocked in stress-depressed states. Injecting *α*_2_-adrenergic receptor agonist intra-clonidine reduced the frequency of neuronal firing and reversed the animals' depressed state [36]. The antiepileptiform effect of MA was blocked by the *α*_2_-adrenergic receptor antagonist [14]. So, MA may act on *α*_2_-adrenergic receptors like clonidine. Third, dual-acting antidepressants like serotonin and norepinephrine reuptake inhibitors (SNRIs) as well as norepinephrine and dopamine reuptake inhibitors (NDRIs) have better efficacy than one system because of the multisystem monoaminergic pathway. They are the primary choices for clinicians in reducing residual symptoms and remission [37, 38]. MA exerted an analgesic effect through both the adrenergic system and the serotonin system. It is also possible to exert antidepressant effects through these two systems.

Moreover, the lack of monoamines is thought to be the leading cause of major depressive disorder (MDD), and some antidepressants work by increasing monoamine levels in the brain [39]. Representative antidepressants such as reboxetine [40], atomoxetine [41], and nortriptyline [42] inhibit noradrenergic transporters and increase norepinephrine in the brain. It has been reported that MA may promote the release of norepinephrine from neurons [24]. The neurotransmitter norepinephrine plays an important role in cognition, behavior, stress responses, and vigilance [43–45]. When the neurogenesis of the adult animal's hippocampus is destroyed by irradiation, the behavioral effects of antidepressants disappear [46]. Depression leads to atrophy of hippocampal neurons. Antidepressants enhance hippocampal neurogenesis [47–49] and reverse hippocampal volume shrinkage as well as hippocampal neuron loss [39]. NA greatly increased the dentate gyrus-derived neural precursor cells (NPCs) proliferation by activating the *β*_2_-adrenergic receptor [50]. Jhaveri et al. reported that an increased amount of NA activated the neurogenic precursors and stem cells via *β*_3_-adrenergic receptors [51]. Increasing norepinephrine by antidepressant promotes hippocampal neurogenesis through augmenting the survival and differentiation of new granule cells (DG) [52]. MA may increase hippocampal neurogenesis by norepinephrine acting on *β*-adrenergic receptors.

**Figure 4:**
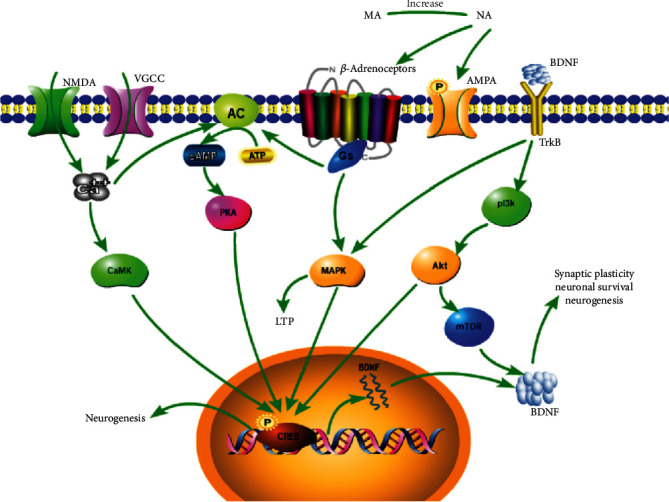
Role of *β*-adrenoceptors on antidepressant effect. All arrows indicate activation arrows. Gs: stimulating adenylate cyclase g protein; ATP: adenosine triphosphate; AC: adenylate cyclase; cAMP: cyclic adenosine monophosphate; PKA: protein kinase A; CREB: cAMP-response element-binding protein; BDNF: brain-derived neurotrophic factor; LTP: long-term potentiation; AMPA: *α*-amino-3-hydroxy-5-methyl-4-isoxazole propionate receptor.

Finally, injection of norepinephrine or isoproterenol intracerebroventricularly (i.c.v.) enhanced analgesic effects of MA, and this effect was attenuated by *β*-adrenoceptors antagonist [17, 26]. MA seems to produce an analgesic effect by activating the *β*-adrenoceptors. The *β*-adrenoceptors can activate Gs. And then Gs activates adenylate cyclase (AC) to produce cyclic adenosine monophosphate (cAMP). cAMP further promotes phosphorylation of cAMP response element-binding (CREB), which in turn produces brain-derived neurotrophic factor (BDNF) [53] (Figure 4). Norepinephrine activated AC through *β*-adrenoceptors and increased the level of cAMP [25]. It is consistent with the previous description that MA promoted the release of norepinephrine. Moreover, some antidepressants act on CREB/BDNF pathway [54–57]. Increased expression of BDNF is considered to be an important mechanism of synaptic plasticity [58–60] and neurogenesis [61–63]. CREB plays an important role in neurogenesis and in reducing depressive symptoms in mice [64]. *β*_3_-adrenergic receptor agonists reduced the immobility time of mice in forced swimming tests. The increase of NA by norepinephrine reuptake inhibitor in the synaptic cleft increased BDNF expression in the dentate gyrus (DG) of the hippocampus through *β*_3_-adrenoceptor [39]. It seems that the antidepressant effects require the activation of *β*_3_-adrenergic receptors. MA may activate *β*_3_-adrenoceptor through norepinephrine (Figure 4).

Synaptic plasticity is currently considered to be an important basis for the formation of learning and memory. It is the fastest-growing research field in neuroscience. Synaptic plasticity includes structural plasticity and functional plasticity. The plasticity of the structure refers to the change in synaptic morphology and quantity caused by repeated synaptic activity. Functional plasticity refers to changes in synaptic transmission efficiency, including long-term potentiation (LTP) and long-term depression (LTD) [65]. Various forms of stress impair long-term potentiation and enhance long-term inhibition [66–69]. Tricyclic antidepressants increase synaptic plasticity at different levels [70]. Norepinephrine facilitates or induces LTP of the population spike through the *β*-adrenergic receptor [53]. Activation of *β*-adrenergic receptors increases the concentration of the cAMP, followed by activation of protein kinase A(PKA) and mitogen-activated protein kinase (MAPK) [71]. Also, norepinephrine-induced long-term potentiation may require activation of N-methyl-D-aspartic acid receptor (NMDA) receptors [72]. Calcium ions enter the cell through the activated NMDA receptors, then bind to intracellular calmodulin and activate adenylate cyclase to increase cAMP levels [73]. Activation of adenylate cyclase, PKA, and MAPK is involved in long-term potentiation induction [74–77]. Besides, norepinephrine reduces the threshold of LTP through phosphorylation of AMPA receptor subunit GluR1 [78]. Therefore, MA may affect synaptic plasticity through increasing norepinephrine. The possible mechanisms of the antidepressant effects induced by MA are outlined in Figure 4.

## 3. Side Effects of MA

Mesaconitine, a diester-diterpenoid alkaloid in aconite roots, is considered to be one of the most important bioactive ingredients and toxic ingredients [79]. Understanding the toxicity and toxicokinetic of MA is important for the application of MA and risk control. The therapeutic window of MA is narrow [80]: studies have shown that the median lethal dose (LD_50_) of a single oral administration MA was 1.9 mg/kg in animal [81] and the half-life of MA was around 2.8–5.8 h [82]. The intravenous LD_50_ value in mice was 0.068 mg/kg [83]. Data from toxicological tests for MA have been presented by Zhou et al. [84] (Table 2). Although there are few reports on the toxicity of MA, three major diterpenoid alkaloids aconitine (AC), MA, and hypaconitine (HA) may share similar cardiotoxicity and mechanisms because of similar core structures [79]. Aconitine-type alkaloids are unstable and unsafe [28, 84–86]. Studies on the metabolism of MA in organisms have been reported [10, 87]. However, a comprehensive MA metabolism database still needs to be built for future pharmacological studies and clinical use [80]. In order to better understand the toxicity of MA, other advanced methods like an electrocardiogram, histopathology, serum biomarkers, and lipidomic profile changes need to be applied [79].

The diester diterpene alkaloids (DAs) with acetyl group at the C-8 position and ester group the C-14 benzoyl are toxic [84]. As the ester bond is hydrolyzed, the toxicity of MA is reduced [83, 87]. However, its pharmacological activity has not been affected [84]. Therefore, it is possible to improve the biological and pharmaceutical properties of MA through structural modification [19].

## 4. Conclusion

MA exerted analgesic effects and antidepressant effects through the serotonin system. Besides, MA exerted analgesic and antiepileptiform effects through the noradrenergic system. Like norepinephrine reuptake inhibitors and tricyclic antidepressants, MA can also increase norepinephrine levels, possibly through norepinephrine acting on related targets to produce multiple neuropharmacological effects. Therefore, the pharmacological effect of MA on depression is worthy of further study. Moreover, a thorough understanding of the toxicity and toxicokinetics of MA is required through advanced methods.

## Figures and Tables

**Table 1:** Experimental information of the analgesic effect of MA.

Animal	Route	Most intense analgesia	ED_50_ values	Mode of action	Medicine	Method	References
Male mice (20-24 g) of the Std:ddY strain Male rats (200-220 g) of the Wistar strain.	s.c. MA (60 *μ*g/kg) s.c. MA (20 *μ*g/kg) i.c.	40 min after s.c	28 *μ*g/kg (95% confidence limit: 11-37) in acetic acid-induced writhing 11 *μ*g/kg (4-28) in tail flick	Dose-dependent and time-dependent	Mesaconitine hydrobromide	Acetic acid-induced writhing tail-flick	[26]....

Male Sprague-Dawley rats (250-350 g)	Intracerebral cannulation 10 ng/rats into NRPG 5 ng/rats into NRM 5 ng/rats into PAG	10 min 5 min 5 min		Dose-dependent and time-dependent	Mesaconitine hydrobromide	Paw pressure test	[22]....

Male rats of the Sprague-Dawley strain (200-250 g)	Intracerebral cannulation 50 and 100 ng/rat into the NRPG 50 ng/rat into the NRGC 50 ng/rat into the PAG 0.5 and 1.0 *μ*g/rat into the lumbar enlargement	20 to 120 min 20 to 120 min 5 to 120 min 10 to 60 min	9.1 *μ*g/kg- (95% confidence limits: 5.1-16.2) intravenously	Dose-dependent and time-dependent	Mesaconitine hydrobromide	Tail immersion test	[17]....

Male NMRI mice (25-30 g)	i.v.		0.025 (0.021-0.034)	Dose-dependent and time-dependent	Mesaconitine	Formalin test	[18]....

Abbreviations: MA: mesaconitine; NRPG: nucleus reticularis paragigantocellularis; NRM: nucleus raphe magnus; NGF: nerve growth factor; PAG: periaqueductal gray.

**Table 2:** LD_50_ of mesaconitine.

Alkaloid	LD_50_ (mg/kg)			
	p.o.	s.c.	i.p.	i.v.
Mesaconitine	1.90 (mice)	0.20–0.38 (mice) 0.204 (rat)	0.20–0.30 (mice)	0.068–0.13 (mice)

## Abbreviations

BDNF: Brain-derived neurotrophic factor
CREB: cAMP-response element-binding protein
NRPG: Nucleus reticularis paragigantocellularis
NRM: Nucleus raphe magnus
NGF: Nerve growth factor
PAG: Periaqueductal gray
BM: Benzoylmesaconine
cAMP: Cyclic adenosine monophosphate
LD50: Median lethal dose
NA: Noradrenaline
PKA: Protein kinase A
MAPK: Mitogen-activated protein kinase
LTP: Long-term potentiation
NMDA: N-methyl-D-aspartic acid receptor.
